# Micro-RNA-125a mediates the effects of hypomethylating agents in chronic myelomonocytic leukemia

**DOI:** 10.1186/s13148-020-00979-2

**Published:** 2021-01-06

**Authors:** Johannes Lorenz Berg, Bianca Perfler, Stefan Hatzl, Marie-Christina Mayer, Sonja Wurm, Barbara Uhl, Andreas Reinisch, Ingeborg Klymiuk, Sascha Tierling, Gudrun Pregartner, Gerhard Bachmaier, Andrea Berghold, Klaus Geissler, Martin Pichler, Gerald Hoefler, Herbert Strobl, Albert Wölfler, Heinz Sill, Armin Zebisch

**Affiliations:** 1 Division of Hematology, Medical University of Graz, Auenbruggerplatz 38, 8036 Graz, Austria; 2 Core Facility Molecular Biology, Medical University of Graz, Graz, Austria; 3 Department of Genetics, University of Saarland, Saarbrücken, Germany; 4 Institute for Medical Informatics, Statistics and Documentation, Medical University of Graz, Graz, Austria; 5 5th Medical Department with Hematology, Oncology and Palliative Medicine, Hospital Hietzing, Vienna, Austria; 6 Sigmund Freud University, Vienna, Austria; 7 Division of Oncology, Medical University of Graz, Graz, Austria; 8 Department of Experimental Therapeutics, The University of Texas MD Anderson Cancer Centre, Houston, TX USA; 9 Diagnostic and Research Institute of Pathology, Medical University of Graz, Graz, Austria; 10 Otto Loewi Research Centre, Immunology and Pathophysiology, Medical University of Graz, Graz, Austria; 11 Otto-Loewi Research Centre for Vascular Biology, Immunology and Inflammation, Division of Pharmacology, Medical University of Graz, Universitätsplatz 4, 8010 Graz, Austria

**Keywords:** Chronic myelomonocytic leukemia, Hypomethylating agent, Azacitidine, miRNA, Tumor suppressor

## Abstract

**Background:**

Chronic myelomonocytic leukemia (CMML) is an aggressive hematopoietic malignancy that arises from hematopoietic stem and progenitor cells (HSPCs). Patients with CMML are frequently treated with epigenetic therapeutic approaches, in particular the hypomethylating agents (HMAs), azacitidine (Aza) and decitabine (Dec). Although HMAs are believed to mediate their efficacy via re-expression of hypermethylated tumor suppressors, knowledge about relevant HMA targets is scarce. As silencing of tumor-suppressive micro-RNAs (miRs) by promoter hypermethylation is a crucial step in malignant transformation, we asked for a role of miRs in HMA efficacy in CMML.

**Results:**

Initially, we performed genome-wide miR-expression profiling in a *Kras*^*G12D*^-induced CMML mouse model. Selected candidates with prominently decreased expression were validated by qPCR in CMML mice and human CMML patients. These experiments revealed the consistent decrease in miR-125a, a miR with previously described tumor-suppressive function in myeloid neoplasias. Furthermore, we show that miR-125a downregulation is caused by hypermethylation of its upstream region and can be reversed by HMA treatment. By employing both lentiviral and CRISPR/Cas9-based miR-125a modification, we demonstrate that HMA-induced miR-125a upregulation indeed contributes to mediating the anti-leukemic effects of these drugs. These data were validated in a clinical context, as miR-125a expression increased after HMA treatment in CMML patients, a phenomenon that was particularly pronounced in cases showing clinical response to these drugs.

**Conclusions:**

Taken together, we report decreased expression of miR-125a in CMML and delineate its relevance as mediator of HMA efficacy within this neoplasia.

## Background

Chronic myelomonocytic leukemia (CMML) is an aggressive hematopoietic neoplasia, which is caused by malignant transformation of hematopoietic stem and progenitor cells (HSPCs) [1]. It combines both myeloproliferative and myelodysplastic features and is therefore classified as a myelodysplastic syndrome/myeloproliferative neoplasia (MDS/MPN) overlap syndrome [1]. CMML has an inherent risk of acute myeloid leukemia (AML) transformation, particularly in cases classified as high-risk situations in the currently used risk-stratification tools [2, 3]. Although some patients can be cured with high-dose chemotherapy and allogeneic hematopoietic stem cell (HSC) transplantation, the majority are diagnosed at an older age and therefore ineligible for this therapeutic approach [2, 3]. The current gold standard for this patient collective are the hypomethylating agents (HMAs), azacitidine (Aza) and decitabine (Dec) [3, 4]. HMAs act via inducing direct cytotoxicity on the one hand and via hypomethylation of DNA on the other hand. Particularly, DNA hypomethylation is thought to restore the expression of silenced tumor-suppressor genes, which in turn potentiates the anti-leukemic effects [5, 6]. Unfortunately, the HMA target genes, which mediate the anti-leukemic effects of these drugs, are largely unexplored. Knowledge of these effectors could potentially help predict the success or even increase the efficacy of HMAs in CMML and other neoplasias.

Micro-RNAs (miRs) are small, noncoding RNA fragments and play a central role in the regulation of gene expression profiles [7, 8]. Aberrant expression profiles of miRs have been described in a broad selection of human tumors, and their functional relevance for malignant transformation is well established nowadays [9, 10]. miRs have also been studied in myeloid neoplasias. In this context, they are functionally relevant for leukemogenesis on the one hand and for prognostic risk stratification on the other hand [6, 11, 12]. Of interest, the silencing of tumor-suppressive miRs in myeloid neoplasias is often caused by hypermethylation of the respective upstream/promoter regions. Consequently, HMAs can reverse this miR downregulation in some cases, which has been shown to contribute to the anti-leukemic properties of these drugs [5, 6]. Unfortunately, however, the knowledge about miRs in CMML is scarce.

We hypothesized that the expression of tumor-suppressive miRs is decreased in CMML and that this downregulation is relevant for HMA treatment. Therefore, we performed miR expression profiling in murine and human CMML specimens and identified a consistent downregulation of the tumor-suppressive miR-125a. We could further show that decreased miR-125a expression is caused by hypermethylation of its upstream/promoter region and can be restored by treatment with HMAs. Finally, we show that the anti-leukemic efficacy of these drugs is at least partly mediated via increasing the expression of miR-125a.

## Results

### miR-125a expression is decreased in CMML

**Fig. 1:**
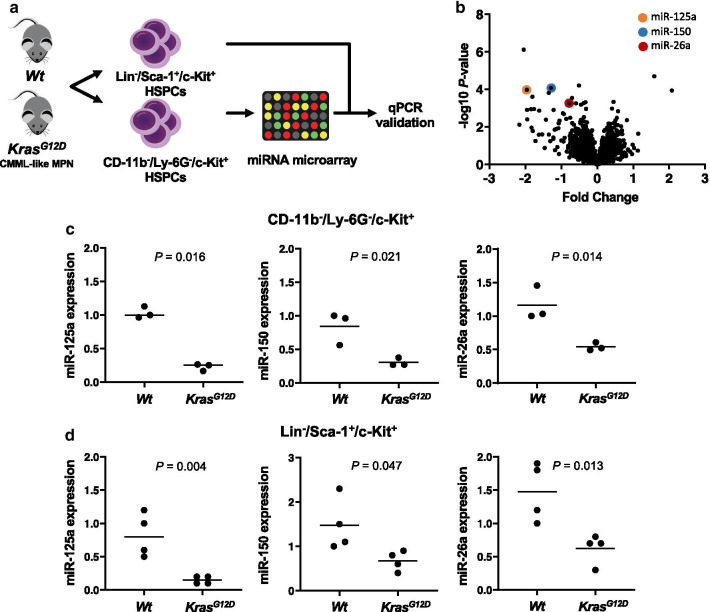
Deregulation of miR expression levels in a murine model of CMML. **a** To screen for aberrant miR expression profiles in CMML, we performed miR-microarray analysis in CD-11b^−^/Ly-6G^−^/c-Kit^+^ HSPCs isolated from the bone marrow of mice with a *Ras*-induced CMML-like MPD. Therefore, four *Mx1-Cre*^+^*/Kras*^*G12D*^ mice and four *Kras*^*Wt*^ control mice were injected with pIpC as outlined in the materials and methods section. Mice were killed and analyzed 6 weeks later. The development of the CMML-like MPD at this time was verified as outlined in Additional file 1: Fig S13. **b** Volcano plot showing deregulated miRs in CMML-HSPCs of *Mx1-Cre*^+^*/Kras*^*G12D*^-mutated mice. The horizontal axis depicts the x-fold expression change in *Mx1-Cre*^+^*/Kras*^*G12D*^-mutated animals and transformed *P*-values are depicted on the vertical axis. miRs with higher expression in *Kras*^*G12D*^-mutated HSPCs are displayed on the right, whereas miRs with decreased expression levels in *Kras*^*G12D*^-mutated HSPCs are displayed on the left. **c**–**d** qPCR validation of the miR microarray results shows decreased miR-125a, miR-150, and miR-26a expression in CD-11b^−^/Ly-6G^−^/c-Kit^+^ (**c**, n = 3) and Lin^−^/Sca-1^+^/c-Kit^+^ (**d**, n = 4) HSPC compartments of *Mx1-Cre*^+^*/Kras*^*G12D*^ mice. At least three mice per group were analyzed. The dot plots show the relative miR expression with the median indicated as horizontal line. One *Kras*^*Wt*^ mouse was always used as a calibrator and its expression is set to 1. Group differences were assessed by t test. Wt, wildtype;

We initially aimed to identify miRs with decreased expression in CMML. Therefore, we performed miR-microarray expression profiling, covering 1908 miRs, in a transgenic *Kras*^*G12D*^-driven mouse model of CMML-like myeloproliferative disease (MPD) [13, 14]. Although CMML is thought to arise from HSCs and early HSPCs, [15], we initially focused on CD-11b^−^/Ly-6G^−^/c-Kit^+^ myeloid progenitor cells, which was a necessary step to produce sufficient amounts of RNA for microarray experiments (Fig. 1a). Interestingly, these analyses revealed extensive deregulation of miR expression profiles in myeloid progenitors of CMML-like MPD, with the majority showing decreased expression in the leukemia samples (Fig. 1b and Table 1).

**Table 1:** TOP10 downregulated miRs in CMML-MPDs of *Kras*^*G12D*^ mice. miRs were ranked according to their x-fold expression change in *Kras*^*G12D*^ mice as compared to the wildtype controls. Only statistically significant miRs with a mean signal intensity of > 4 on all arrays were selected

Downregulated miRs			
ID	Accession #	Fold change	*p value*
mmu-miR-99b-5p	MIMAT0000132	-4.13	< 0.001
mmu-miR-125a-5p	MIMAT0000135	-3.89	< 0.001
mmu-miR-130a-3p	MIMAT0000141	-3.59	0.001
mmu-miR-146a-5p	MIMAT0000158	-3.49	< 0.001
mmu-miR-99a-5p	MIMAT0000131	-3.06	0.001
mmu-miR-676-3p	MIMAT0003782	-2.55	< 0.001
mmu-miR-150-5p	MIMAT0000160	-2.44	< 0.001
mmu-let-7e-5p	MIMAT0000524	-2.32	0.001
mmu-miR-26a-5p	MIMAT0000082	-1.71	0.001
mmu-miR-3535	MIMAT0031410	-1.63	< 0.001

We then selected a series of miRs for further analyses according to the following criteria: i) the miR was amongst the TOP10 downregulated miRs and ii) a tumor-suppressive role was proven in myeloid leukemogenesis previously. Initially, we successfully validated the downregulation of the selected miR candidates (miR-125a, miR-150, and miR-26a) [16–19] by qPCR (Fig. 1c). Next, we were interested in whether downregulation of these miRs truly occurs at the HSC level. In this respect, it has to be noted that we used CD-11b^−^/Ly-6G^−^/c-Kit^+^ myeloid progenitors for our miR-transcriptome profiling in mice. Therefore, we additionally analyzed Lin^−^/Sca-1^+^/c-Kit^+^ (LSK) cells, which is a population enriched for HSCs [20]. The decreased expression of all three miRs could be validated within the LSK compartment (Fig. 1d), which indicates that their downregulation occurs at the HSC level.

**Fig. 2:**
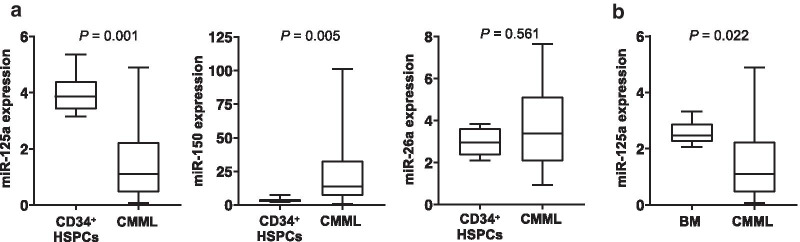
miR-125a expression is decreased in primary CMML patient specimens. **a** The box plots show the relative miR-125a, miR-150, and miR-26a expression levels in 36 CMML patient specimens compared to six CD34^+^ HSPCs. **b** The relative expression level of miR-125a in 36 CMML patients compared to six whole BM specimens of healthy donors and patients with lymphatic diseases without BM affection is displayed. The graphs denote the miR expression normalized to a calibrator, set to 1 (U937 for miR-125a and miR-26a; GDM-1 for miR-150). Differences between the groups were assessed with the Mann–Whitney U test. CMML, chronic myelomonocytic leukemia; BM, bone marrow; HSPCs, hematopoietic stem and progenitor cells

We then were interested in whether these findings can be translated into human CMML. We, therefore, analyzed 36 primary CMML patient specimens (for clinical characteristics, see Additional file 1: Table 1) and compared the results to six healthy CD34^+^ HSPCs. In these assays, only miR-125a demonstrated a significantly decreased expression in the CMML specimens studied (Fig. 2a). It was previously shown that miR-125a is high in HSPCs and decreases during the physiologic myeloid differentiation [21]. As we compared whole bone marrow (BM) of CMML patients with a purified population of CD34^+^ HSPCs, our analysis might have been biased by the presence of differentiated cells within the CMML cases. To exclude such a scenario, we analyzed miR-125a expression in whole BM specimens of healthy donors and patients with lymphatic diseases without BM affection (*n* = 6). Again, miR-125a expression within these samples was significantly higher than in the CMML cases (Fig. 2b). Of note, decreased expression of miR-125a was not associated with a specific clinical or molecular feature of CMML (Additional file 1: Table 1, Additional file 1: Figs. S1–S2).

### Decreased miR-125a expression is caused by hypermethylation of its upstream/promoter region and can be increased by HMA treatment

**Fig. 3:**
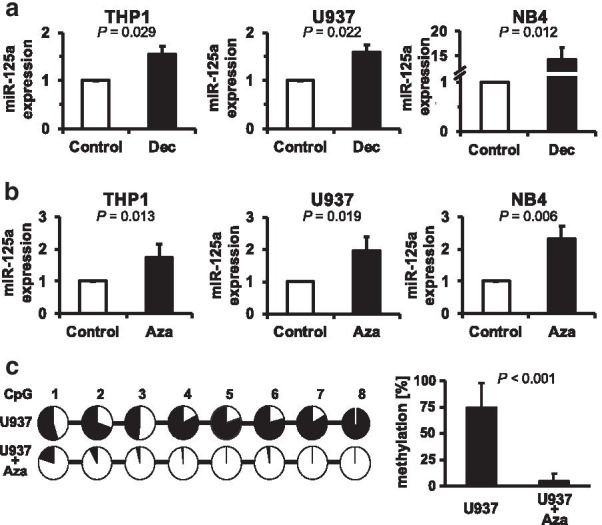
Decreased miR-125a expression is caused by hypermethylation of its upstream/promoter region and can be increased by HMA treatment. **a** miR-125a expression was assessed by qPCR in the myeloid cell lines THP1, U937, and NB4 after treatment with 5 µM decitabine for 48 h. For comparison of the different conditions, respective control situations (treated with the empty dissolvent only) were set at a value of 1. The relative increase in miR-125a expression in the decitabine-treated conditions was calculated as the ratio of decitabine-treated to control-treated expression levels. **b** miR-125a expression after treatment with 2.5 µM azacitidine for 24 h. The relative increase in miR-125a expression in the azacitidine-treated conditions compared to controls is displayed. Graphs denote the mean ± SD of at least three independent experiments. Comparisons against the control condition were performed using a one-sample t test against a reference value of 1. Of note, the increase in miR-125a expression could also be observed after incubation with a lower azacitidine concentration (1 µM; Additional file 1: Fig. S4) **c** Eight CpG-sites within a CpG-rich region upstream of miR-125a [23] were studied by bisulfite sequencing in U937 cells before and after treatment with 2.5 µM azacitidine for 24 h. This region roughly correlates to the miR-125a promoter region [52]. The panel on the left demonstrates the percentage of methylated sequencing reads for each CpG site studied; the panel on the right depicts the mean percentage of methylation across all eight CpG sites ± SD. Statistical differences were assessed with the t test. Aza, azacitidine; Dec, decitabine

**Fig. 4:**
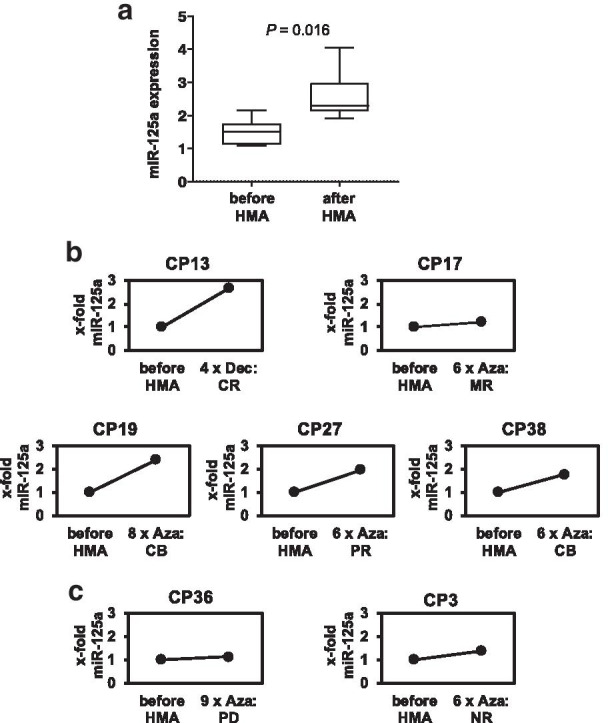
miR-125a expression increases in CMML patients after the therapy with HMAs. **a** Box plot depicting the miR-125a expression levels in seven paired CMML patient specimens collected before and after treatment with HMAs. miR-125a expression was analyzed by qPCR and displayed as the log-transformed x-fold expression of the calibrator (U937 cells). Differences between expression values before and after treatment were assessed with the paired Wilcoxon signed-rank test. **b**,**c**, miR-125a expression levels in individual CMML patients with (**b**) or without (**c**) clinical response to HMA therapy. miR-125a expression levels assessed before HMA treatment were used as controls and set to 1. miR-125a levels after HMA treatment are displayed as x-fold change to the respective control sample. HMA hypomethylating agent; CP, CMML patient, Dec, decitabine; Aza, azacitidine, CR, complete response; MR, marrow response; CB, clinical benefit; PR, partial response; PD, progressive disease; NR, no response

We then asked whether the decreased expression of miR-125a is caused by hypermethylation of its upstream/promoter region. Recent work has pinpointed the role of hypermethylation of the miR-125a upstream region as the mechanism behind miR-125a downregulation [22–25]. To test this hypothesis, we employed a series of leukemia cell lines with decreased miR-125a expression (THP1, U937, NB4, Additional file 1: Fig. S3) and treated these cells with the HMAs Aza and Dec, respectively. Importantly, both substances significantly increased the expression of miR-125a in all cell lines tested (Fig. 3a,b, Additional file 1: Fig. S4). These data suggest that the decrease in miR-125a expression in myeloid leukemias is caused by hypermethylation and is reversible by HMA treatment. To test this assumption, we performed bisulfite sequencing of the miR-125a upstream/promoter region in U937 cells before and after HMA treatment. Indeed, HMA treatment significantly decreased the methylation content within the upstream region studied (Fig. 3c). To delineate whether such an HMA-induced increase in miR-125a expression can also be observed in HMA-treated CMML patients, we analyzed the miR-125a expression in seven paired CMML patient specimens obtained before and after treatment with Aza or Dec. In agreement with our in vitro data, we thereby observed a statistically significant increase in miR-125a expression after HMA treatment (Fig. 4a). Interestingly, the increase in miR-125a expression was particularly pronounced in the five patients presenting with clinical response to HMA therapy (Fig. 4b; response assessment according to the recently published response criteria for MDS/MPN in adults [26]). Unfortunately, bisulfite sequencing in patient specimens failed due to technical reasons. Therefore, we performed a database analysis via the National Center for Biotechnology Information (NCBI)’s Gene Expression Omnibus (GEO; https://www.ncbi.nlm.nih.gov/geo/; GSE40870). In this study, the authors performed genome-wide DNA methylation profiling in myeloid leukemia patient specimens treated with Dec and cytarabine, respectively, and compared the results to control-treated samples [27]. We re-analyzed the methylation of a specific CpG-site within the miR-125a upstream/promoter region and analyzed the methylation change to control-treated samples. Importantly, Dec caused significant demethylation of the miR-125a upstream/promoter region, whereas the classical chemotherapeutic agent cytarabine failed to do so (Additional file 1: Fig. S5). These data further support our observation that the HMA-induced increase in miR-125a expression is caused by demethylation of its upstream/promoter region.

### The anti-leukemic effects of HMAs are partly mediated by increasing the expression of miR-125a

**Fig. 5:**
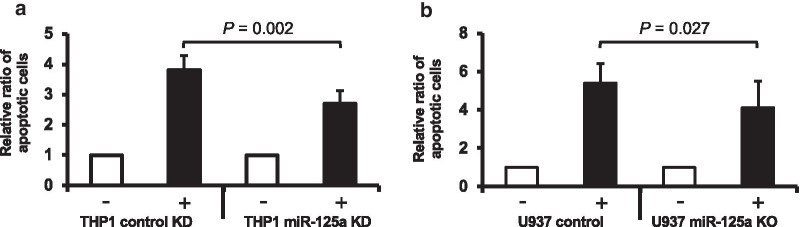
The anti-leukemic effects of HMAs are partly mediated by increasing the expression of miR-125a. **a** THP1 cells were transiently transfected with a miR-125a-specific hairpin inhibitor (THP1 miR-125a KD) and scrambled control (THP1 control KD), respectively. **b** miR-125a was deleted in U937 by employing the CRISPR/Cas9 technology (U937 miR-125a KO); parental U937 cells were used as controls. Apoptosis was measured after 24-h treatment with Aza (2.5 µM for THP1, 5 µM for U937; indicated as +) or empty dissolvent (indicated as −) by Annexin-V/7AAD assays. The respective control situations (treated with the empty dissolvent only) were set at a value of 1, and the relative increase in apoptosis in the Aza-treated conditions was calculated as the ratio of Aza-treated to control-treated cells. The graphs represent the mean ± SD of at least three independent experiments. Statistical significance was evaluated using a paired Student’s *t* test

As described above, clinical data indicate that HMA treatment increases the expression of downregulated miR-125a in CMML patients and suggest a potential link between the rise of miR-125a and the clinical efficacy of HMAs. Therefore, we aimed to delineate whether some anti-leukemic HMA effects might be mediated via the increase in miR-125a expression. We initially tested the efficacy of Aza, the most employed HMA, in the above-mentioned monocytic leukemia cell lines. In agreement with the published literature, this caused the induction of cell death, which was mediated via an increase in apoptosis (Additional file 1: Figs. S6–S7) [28, 29]. Aza thereby mimicked the effects of miR-125a overexpression, which increased the apoptosis within these cells as well (Additional file 1: Fig. S8). We then employed THP1 cells with a hairpin inhibitor-mediated knockdown of miR-125a (Additional file 1: Fig. S9). As this knockdown had been established by antisense oligonucleotides, it cannot be rescued by the treatment with HMAs. Indeed, Aza failed to increase the expression of miR-125a in these cells (Additional file 1: Fig. S10). Most importantly, the effects of Aza on apoptosis were significantly diminished in this situation (Fig. 5a), which indicates that, indeed, some anti-leukemic effects of HMAs are mediated by increasing miR-125a expression. To further corroborate these results, we repeated these experiments in U937 cells. As hairpin inhibitor-mediated miR-125a knockdown was unsuccessful in this cell line, we completely deleted miR-125a by employing the CRISPR/Cas9 technology (Additional file 1: Fig S9). In agreement with the results from THP1, miR-125a deletion reduced the sensitivity to Aza (Fig. 5b). Finally, we also tested the effects of Aza treatment in THP1 cells, where miR-125a expression was already increased by stable transfection with a miR-125a expression construct. In this situation, Aza treatment increased the miR-125a expression further, suggesting a synergistic or additive effect (Additional file 1: Figure S11). Indeed, Aza treatment in miR-125a-overexpressing cells caused a further increase in apoptosis when compared with miR-125a-overexpressing cells treated with empty dissolvent (Additional file 1: Figure S12).

## Discussion

In this study, we aimed to delineate the potential mediators of HMA treatment response in CMML. We initially screened for a potential downregulation of tumor-suppressive miR expression profiles in CMML by applying a miR-microarray platform covering more than 1900 miRs. By employing a genetically homogenous *Kras*^*G12D*^-induced murine model of CMML, we were able to identify extensive deregulation of miR expression profiles, with the majority of these miRs being downregulated in CMML-HSPCs. By additionally analyzing 36 primary CMML patient specimens, we were able to validate miR-125a as one of the most prominently downregulated miRs in CMML, underlining the relevance of our mouse model for the human setting. Aberrant expression of miR-125a has been described in a variety of myeloid malignancies and proved to exhibit a tumor-suppressive function during myeloid leukemogenesis [16, 30]. Indeed, transgenic deletion of miR-125a in mice caused the development of MPDs and urogenital abnormalities [16]. These data are corroborated by in vitro data of the current study, where miR-125a overexpression in monocytic leukemia cells with decreased endogenous miR-125a had a pronounced anti-leukemic effect (Additional file 1: Fig S8). Interestingly, Tatsumi and co-workers reported that mice with a complete deletion of miR-125a developed a less severe MPD phenotype than animals with a heterozygous deletion; the latter showing a decreased but still measurable miR-125a expression. The authors thereby demonstrated compensatory inhibitory pathways that only became active when a complete deletion of miR-125a occurred. The role of miR-125a is further complicated by the fact that oncogenic properties were described in the context of myeloid leukemogenesis as well [21, 31]. Taken together, the currently available functional in vivo data imply that miR-125 has to be finely tuned to keep HSPCs in a physiologic state and that miR-125a deregulation, both by increased and by decreased expression levels, may cause the development of myeloid leukemias. Such a scenario has also been shown for other miRs, including miR-126a, where both overexpression and knockdown enhanced murine *AML1-ETO/RUNX1-RUNX1T1*-driven leukemogenesis [32]. To put these findings in a clinical context, it is necessary to delineate the expression profile of these miRs in primary patient specimens of the respective malignancy. In this study, we now report significantly decreased miR-125a in 36 primary patient specimens of CMML, which suggests that a decrease in miR-125a expression represents the major mechanism of miR-125a deregulation during CMML development and highlights its tumor-suppressive function within this malignancy.

Another interesting finding of our study was that decreased expression of miR-125a is caused by hypermethylation of its upstream/promoter region. These data are in line with results from AML and solid cancers [22–25]. Indeed, we demonstrated that miR-125a expression in myeloid cell lines with low endogenous miR-125a levels was significantly increased by treatment with both Aza and Dec. These findings are also in agreement with data from the primary patient specimens. By qPCR-based miR expression analyses in paired patient specimens obtained before and after/during HMA treatment, we observed that miR-125a expression levels after HMA therapy are significantly higher than before. Interestingly, we observed that the degree of miR-125a increase in HMA-treated patients was particularly pronounced in cases where HMAs demonstrated clinical efficacy. It has to be noted that this clinical observation is limited by the fact that our cohort of sequential samples was too small to corroborate these findings statistically. Additionally, the HMA overall response rate of > 70% within these cases seems to be rather high compared with results from the literature [3]. Hence, a selection bias of these cases cannot be excluded. While larger and prospective cohorts will be necessary to ultimately unravel a potential link between HMA-induced miR-125a increase and HMA efficacy, such an association has been described in a previous publication as well. Solly and co-workers studied miR expression profiles in high-risk MDS (HR-MDS) patients treated with Aza [33]. While they described a decreased expression of miR-125a in BM samples of HR-MDS patients as compared with healthy donors at diagnosis, they also described changes in miR-125a expression during Aza treatment. As seen in our study, the authors observed that miR-125a expression increased the following Aza treatment in patients with a sustained response, whereas the opposite was true for patients with primary or secondary Aza resistance. Based on these data, one might speculate that Aza has the potential to increase the expression of miR-125a and that this miR-125a increase might mediate some of the anti-leukemic effects of these substances. To test this assumption, we treated monocytic leukemia cells with Aza and assessed the anti-leukemic effects by measuring the induction of apoptosis. We then performed additional inactivation of miR-125a by shRNA-mediated knockdown and CRISPR/Cas9-mediated knockout, respectively. This miR-125a silencing inhibited the Aza-induced increase in miR-125a expression. Most importantly, the prevention of miR-125a upregulation reduced the effects of Aza on inducing apoptosis. These data indicate that at least some of the effects of HMAs are mediated via increasing the expression of miR-125a. Finally, it is worth mentioning that Aza treatment, combined with lentiviral miR-125a overexpression, had a synergistic/additive effect in our experiments. This finding is of particular relevance as the development of miR-mimic pharmaceutics is nowadays feasible [34]. It is appealing to imagine a situation where miR-125a mimics might be used to increase the efficacy of HMAs in CMML or to overcome the resistance to these drugs. While this scenario is a very optimistic outlook into a more distant future, our experiments warrant further preclinical work in this direction.

## Conclusions

In conclusion, we describe a biologically relevant decrease in miR-125a expression in CMML, which is caused by hypermethylation of its upstream/promoter region. We further show that the treatment with HMAs can reverse miR-125a downregulation and that at least some of the anti-leukemic efficacy of HMAs is mediated via increasing the expression of miR-125a. These data provide more insight into the mode of action of HMAs in CMML and will aid in optimizing HMA treatment approaches for patients with myeloid malignancies.

## Methods

### Primary CMML patient samples and cell lines

CMML patient samples (peripheral blood or BM), as well as BM specimens from healthy donors and patients with lymphatic diseases without BM infiltration, were collected and stored at the Division of Hematology, Medical University of Graz (MUG), Austria. Additionally, CMML patient specimens from the Austrian Biodatabase for CMML [4, 35] were included in this study. All samples were processed and stored as described in detail previously [36–38]. All samples contained at least 80% myelomonocytic cells as determined by Giemsa–May–Grünwald-stained cytospin preparations. Healthy CD34 + HSPCs were collected from umbilical cord blood specimens (EasySep, STEMCELL Technologies, Vancouver, Canada) as described in detail previously [39]. 293 T, U937, THP1, and NB4 cell lines were obtained from the German National Resource Center for Biological Material (DSMZ, Braunschweig, Germany) and the Core Facility Alternative Biomodels and Preclinical Imaging (Center for Medical Research at the MUG), respectively. Low passage stocks were frozen, and cells were cultivated for less than 6 months after thawing. Moreover, cells were screened by variable number of tandem repeat profiling (VNTR) for authenticity as previously described [39, 40].

### Mouse strains and isolation of murine HSPCs

All mouse experiments were performed on a C57BL/6 strain background. Mice with a *Mx1-Cre*^+^*/Kras*^*G12D*^ genotype and wildtype controls were used for miRNA expression screening. Intraperitoneal injections of polyinosinic polycytidylic acid (pIpC, Sigma-Aldrich, St. Louis, MO, USA; three times 250 µg/d at every alternate day, starting at the age of 30 days) were performed in all mice. For miR expression analysis, mice were killed by cervical dislocation after anesthesia with isoflurane (4%; AbbVie, North Chicago, IL, USA, mixed with 1,5 l/min O_2_) 6 weeks after pIpC injection. CMML-like MPD in *Kras*^*G12D*^-mutated animals was fully developed at this time (Additional file 1: Fig. S13). For miR-microarray and qPCR experiments, Lin^−^/Sca-1^+^/c-Kit^+^ HSCs and CD-11b^−^/Ly-6G^−^/c-Kit^+^ myeloid progenitor cells, respectively, were collected from the BM by lineage depletion and flow cytometry sorting as described in detail previously [39].

### Cell culture, transfection, CRISPR/Cas9-mediated miR-125a deletion, and in vitro treatments

U937, THP1, NB4, and GDM-1 cells were maintained at 37 °C/5% CO_2_ in RPMI-1640; 293 T were cultivated the same way in DMEM (all from Sigma-Aldrich). The medium was enriched with 10% heat-inactivated fetal bovine serum (FBS) and 1X Antibiotic–Antimycotic (Thermo Fischer Scientific, Waltham, MA, USA, including 100U/mL penicillin, 100 mg/mL streptomycin and 0.25 mg/mL amphotericin B). DMEM medium was further supplemented with 1X GlutaMAX (Thermo Fischer Scientific). For overexpression of miR-125a, THP1 were lentivirally transduced with pEZX-MR03-miR-125a (THP1 miR-125a OE) or the respective empty vector control (THP1 control OE; all constructs from GeneCopoeia, Rockville, MD, USA) according to a previously published protocol [39–41]. Selection of stable cells was performed with 1.5 µg/mL puromycin. For miR-125a knockdown experiments, THP1 cells were transfected with miR-125a-specific miRIDIAN hairpin inhibitors (THP1 miR-125a KD) or scrambled controls (THP1 control KD) using DharmaFECT lipofection as previously described [41, 42] (all reagents from Dharmacon, Lafayette, CO, USA). For generation of miR-125a knockouts in U937 cells, the CRISPR/Cas9 technology was employed using a plasmid-based system with two different single-guide RNAs (sgRNAs) targeting the up- and downstream region of miR-125a. sgRNAs were ligated into pX458 plasmids (Addgene, Watertown, MA, USA; ordering number #48138), originally generated from the Zhang lab [43], co-expressing Cas9 from *Streptococcus pyogenes* and EGFP. U937 cells were co-transfected with the generated plasmids using a Neon Nucleofector (Thermo Fischer) according to the manufacturer’s instructions, and EGFP-positive single cells were sorted using the FACSAria III (BD biosciences). Successful miR-125a knockout was validated by Sanger sequencing and qPCR as previously described [42, 44, 45]. Primer and sgRNA sequences are displayed in Additional file 1: Table 2. In vitro treatments were performed with 2.5 µM Aza (PeproTech, Rocky Hill, NJ, USA; dissolved in RPMI), 5 µM Dec (Sigma-Aldrich; dissolved in RPMI), and 0.5 µM staurosporine (Abcam, Cambridge, UK, dissolved in DMSO) as indicated in the respective figures.

### miR-microarray and qPCR expression profiling

RNA isolation was performed using the miRNeasy Micro Kit (Qiagen, Hilden, Germany) according to the manufacturer’s protocol. The quality of RNA was controlled on a Bioanalyzer BA2100 (Agilent; Foster City, CA), and only samples with an RNA integrity number (RIN) greater than 9 were taken. For miR-microarray expression profiling in murine HSPCs, 300 ng of RNA was assessed using the Affymetrix GeneChip miRNA 4.0 (Affymetrix, Santa Clara, CA, USA) according to the manufacturer’s instructions. Following the miRNA database miRBase (version 20), this kit comprises 36353 gene probes, including 1908 murine miRNAs. Affymetrix Expression Console 1.1.2 was employed for the evaluation of internal array controls and preanalysis, while Partek Genomic Suite v6.6 software (Partek Inc; St Louis, MO) was used for data normalization and analysis. All of these processes have been described in more detail previously [40]. Microarray miR expression data have been deposited in the NCBI’s GEO (https://www.ncbi.nlm.nih.gov/geo/) and are accessible through GEO accession number GSE145083. For qPCR experiments, cDNA was generated using the miScript II RT Kit (Qiagen). The expression of miR-125a, miR-150 and miR-26 was assessed on a LightCycler 480 Instrument II (Roche Life Sciences, Penzberg, Germany) using the miScript SYBR Green PCR Kit (Qiagen) and analyzed by employing the ∆∆CT method as previously described [40, 41, 46]. SNORD72, SNORD61, and RNU6b (Qiagen) were used as reference genes. U937 or GDM-1 cells (for all primary human specimens) and untreated/vector-transfected controls, respectively, served as calibrators. Primer sequences are displayed in Additional file 1: Table 2.

### Bisulfite sequencing

Bisulfite conversion from U937 cells was performed using the EpiTect Bisulfite Kit (Qiagen) according to the manufacturer’s instructions. For targeted methylation analysis of the micro-RNA-125a upstream region, 2 µl of bisulfite-treated DNA was used in a nested PCR approach with the KAPA HiFi HotStart Uracil + Kit (Roche, Mannheim, Germany; for primer sequences see Additional file 1: Table 2). Subsequently, PCR products were indexed and prepared for next-generation sequencing according to published procedures [47, 48]. Libraries were sequenced at 8 pM on an Illumina MiSeq Desktop sequencer with v3 600 chemistry and 20% PhiX control DNA (Illumina, San Diego, CA, USA) according to manufacturer’s instructions. Data were analyzed with the BiQ Analyzer HT software [49].

### Analysis of cell growth, proliferation, and apoptosis

Growth curves were performed as previously described [41, 46]; the number of viable cells was assessed on a TC-20 (Bio-Rad, Hercules, CA, USA). Cell cycle progression/proliferation was assessed by flow cytometry after BrdU/7AAD staining using the BrdU Flow Kit (BD Pharmingen, Franklin Lakes, New Jersey, USA) according to the manufacturer’s instructions and as described previously [41, 46, 50, 51]. BrdU incubation was performed at a concentration of 50 µM for 1 h, and an LSR II machine (BD Biosciences) was used for flow cytometry. Apoptosis was assessed by Annexin-V/7AAD assays (BD Biosciences), as described earlier [41, 46, 50, 51], using a CytoFLEX LX flow cytometer (Beckman Coulter, Brea, CA, USA).

### Statistical analyses and database retrieval

Methylation β-values were downloaded via the NCBI’s GEO (https://www.ncbi.nlm.nih.gov/geo/) on 25 September 2020 from the publicly available GSE40870 dataset of Klco and coworkers [27]. The changes in methylation β-values in Dec/cytarabine-treated conditions compared to the respective control-treated conditions were calculated by one-sample *t* test as outlined in more detail in Figure legend S5.

Group differences in miR-125a expression values of primary patients were compared by Mann–Whitney U or Kruskal–Wallis test. The Wilcoxon signed-rank test was employed for comparison of miR-125a expression before and after HMA treatment in paired patient specimens. Spearman’s rank correlation coefficient was used to analyze the correlation of miR-125a with relevant continuous variables within the patient cohort (white blood cell counts, BM blasts, and age). Mouse data and in vitro experiments were analyzed by unpaired, paired, or one-sample *t* test as depicted in detail in the respective figure legends. All analyses were performed in GraphPad Prism, version 8 (San Diego, CA, USA), and R version 3.6.1 (https://www.r-project.org/). All tests were performed two-sided, and a *P* value < 0.050 was considered statistically significant.

## Supplementary information

**Additional file 1.** Supplementary information.

## Data Availability

Microarray miR expression data have been deposited in the NCB’s GEO (https://www.ncbi.nlm.nih.gov/geo/) and are accessible through GEO accession number GSE145083. All other data are available from the corresponding author on reasonable request.

## Abbreviations

AML: Acute myeloid leukemia
AZA: Azacitidine
BM: Bone marrow
CMML: Chronic myelomonocytic leukemia
DEC: Decitabine
DSMZ: German National Resource Center for Biological Material
FBS: Fetal bovine serum
GEO: Gene Expression Omnibus
HMA: Hypomethylating agent
HR-MDS: High-risk myelodysplastic syndrome
HSC: Hematopoietic stem cell
HSPC: Hematopoietic stem and progenitor cells
LSK: Lin^−^/Sca-1^+^/c-Kit^+^ cells
MDS/MPN: Myelodysplastic syndrome/myeloproliferative neoplasia overlap syndrome
miR: Micro-RNA
MPD: Myeloproliferative disease
MUG: Medical University of Graz
NCBI: National Center for Biotechnology Information
pIpC: Polyinosinic polycytidylic acid
RIN: RNA integrity number
sgRNA: Single-guide RNA
VNTR: Variable number of tandem repeat profiling
