# Supplementary material for: Micro-RNA-125a mediates the effects of hypomethylating agents in chronic myelomonocytic leukemia
Source: Clin Epigenetics. 2021 Jan 6;13:1. doi: 10.1186/s13148-020-00979-2 (PMC7789782; doi:10.1186/s13148-020-00979-2)
Supplement: Supplementary file 1 — Additional file 1. Supplementary information. [file 13148_2020_979_MOESM1_ESM.pdf]

Supplementary Information

Micro-RNA-125a mediates the effects of hypomethylating agents in chronic myelomonocytic leukemia

Johannes Lorenz Berg[1], Bianca Perfler[1], Stefan Hatzl[1], Marie-Christina Mayer[1], Sonja Wurm[1], Barbara Uhl[1], Andreas Reinisch[1], Ingeborg Klymiuk[2], Sascha Tierling[3], Gudrun Pregartner[4], Gerhard Bachmaier[4], Andrea Berghold[4], Klaus Geissler[5,6], Martin Pichler[7,8], Gerald Hoefler[9], Herbert Strobl[10], Albert Wölfler[1], Heinz Sill[1] and Armin Zebisch[1,11]

[1]Division of Hematology, Medical University of Graz, Austria; [2]Core Facility Molecular Biology, Medical University of Graz, Austria; [3]Department of Genetics, University of Saarland, Germany; [4]Institute for Medical Informatics, Statistics and Documentation, Medical University of Graz, Austria; [5]5th Medical Department with Hematology, Oncology and Palliative Medicine, Hospital Hietzing, Vienna, Austria; [6]Sigmund Freud University, Vienna, Austria; [7]Division of Oncology, Medical University of Graz, Graz, Austria; [8]Department of Experimental Therapeutics, The University of Texas MD Anderson Cancer Centre, Houston, TX, USA; [9]Diagnostic and Research Institute of Pathology, Medical University of Graz, Graz, Austria; [10]Otto Loewi Research Centre, Immunology and Pathophysiology, Medical University of Graz, Austria; [11]Otto-Loewi Research Centre for Vascular Biology, Immunology and Inflammation, Division of Pharmacology, Medical University of Graz, Austria.

Supplementary Figures

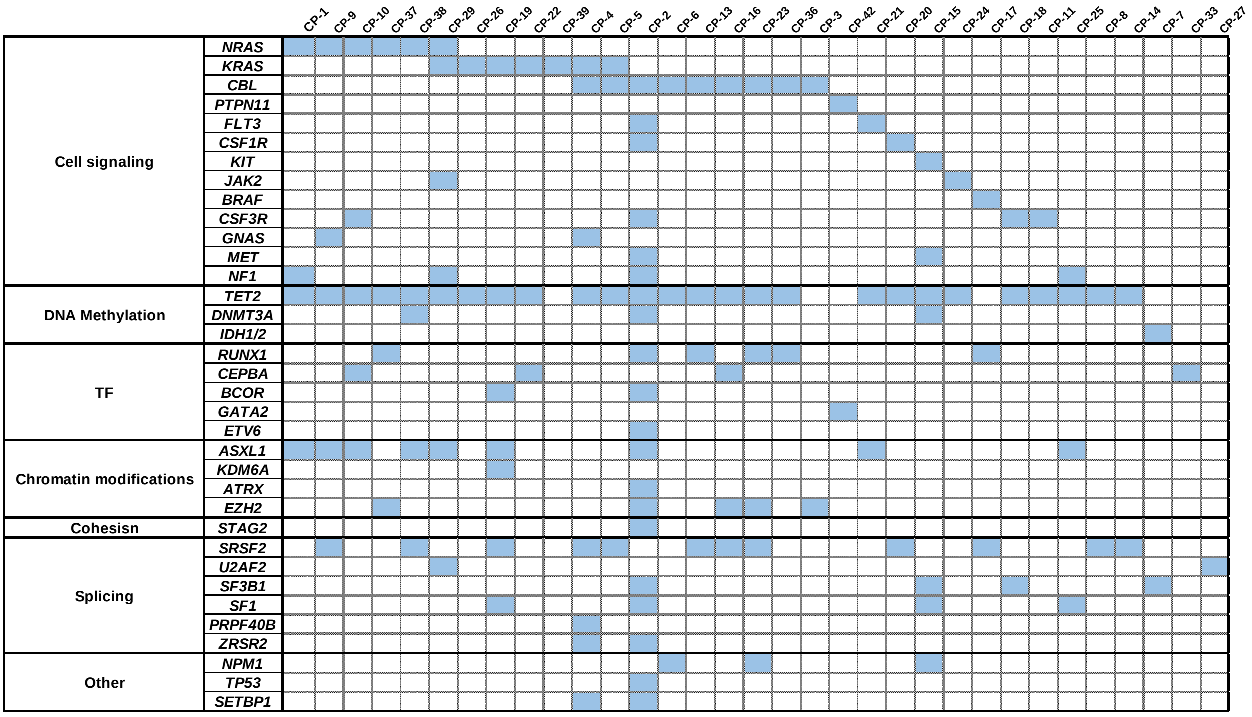

Supplementary Fig S1. Mutational landscape in CMML patients. Next generation sequencing (NGS) results were available in 33/36 CMML patients studied. NGS was performed using an Ion Torrent Sequencing platform, as described previously [1]. The gene list analyzed included 39 genes with recurrent mutations in myeloid neoplasms: *CEBPA* (full coding); *NPM1* (Exon 11); *FLT3* (Exon 14-16,20,21); *ASXL1* (Exon 12); *BCOR* (full coding); *BRAF* (Hotspot Exon 15); *CALR* (Exon 9); *CBL* (Exon 8,9); *CSF3R* (Exon 14-17); *DDX41* (full coding); *DNMT3a* (full coding); *ETNK1* (Exon 3); *ETV6* (full coding); *EZH2* (Exon 16-19); *GATA2* (full coding); *IDH1* (Exon 4); *IDH2* (Exon 4); *JAK2* (Exon 13); *KIT* (Exons 8,10,11,17); *KRAS* (Exon 2,3); *MPL* (Exon 10); *NF1* (full coding); *NRAS* (Exon 2,3); *PHF6* (full coding); *PTPN11* (Exon 3,13); *RUNX1* (Exon 3-8); *SETBP1* (Hotspot Exon 4); *SF3B1* (Exon 14-16); *SF3B2* (full coding); *SFRP1* (full coding); *SRP72* (full coding); *SRSF2* (Hotspot Exon 1); *STAG2* (full coding); *STAT3* (Exon 20,21); *TET2* (Exon 3-11); *TP53* (full coding); *U2AF1* (Exon 2,7,9); *WT1* (Exon 7,9); *ZRSR2* (full coding). Mutation calling necessitated a coverage of at least

1000x, a frequency in the 1000-genome project of <0.01% and a variant allele frequency (VAF) >5%. Only genes with mutations in ≥1 patient(s) are shown.

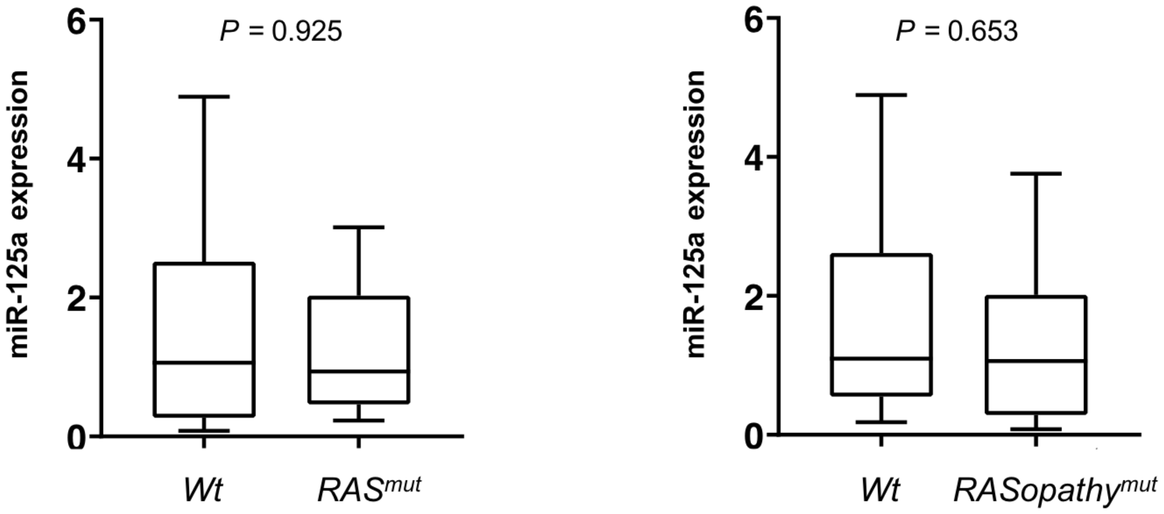

Supplementary Fig S2. Association of miR-125a expression with *RAS* mutations. Box plots displaying miR-125a expression in the 33 CMML patients analyzed by NGS. The left panel depicts patients with (n=12) and without (n=21) mutations in *RAS* (*NRAS* and *KRAS*); patients with (n=21) and without (n=12) *RAS*opathy mutations (*NRAS*, *KRAS*, *NF1*, *CBL*, *PTPN11*) are displayed on the right side of the figure. The graphs denote the miR-125a expression normalized to U937 cells, which were used as a calibrator in all qPCR experiments, and which were set to 1. Differences between the groups were assessed with the Mann-Whitney U test. Wt, Wildtype.

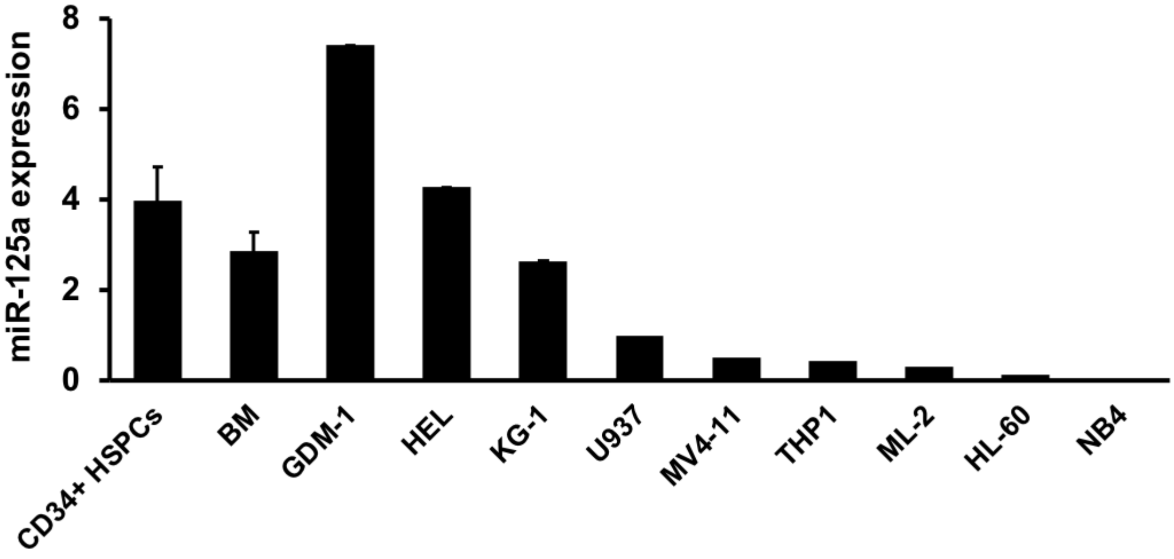

Supplementary Fig S3. THP1 cells exhibit decreased expression of miR-125a. To choose a suitable model cell line for lentiviral overexpression of miR-125a, we assessed the miR-125a expression in nine myeloid leukemia cell lines by qPCR. The myelomonocytic cell line THP1 shows a prominent decrease in miR-125a expression compared to $CD34^+$ HSPCs (n = 6) and healthy BM aspirates (n = 6). The graphs denote the x-fold miR-125a expression of U937 cells, which were used as a calibrator and which were set to 1. Bars for $CD34^+$ HSPCs and BM indicate the mean +/- SD. HSPCs, hematopoietic stem and progenitor cells; BM, bone marrow.

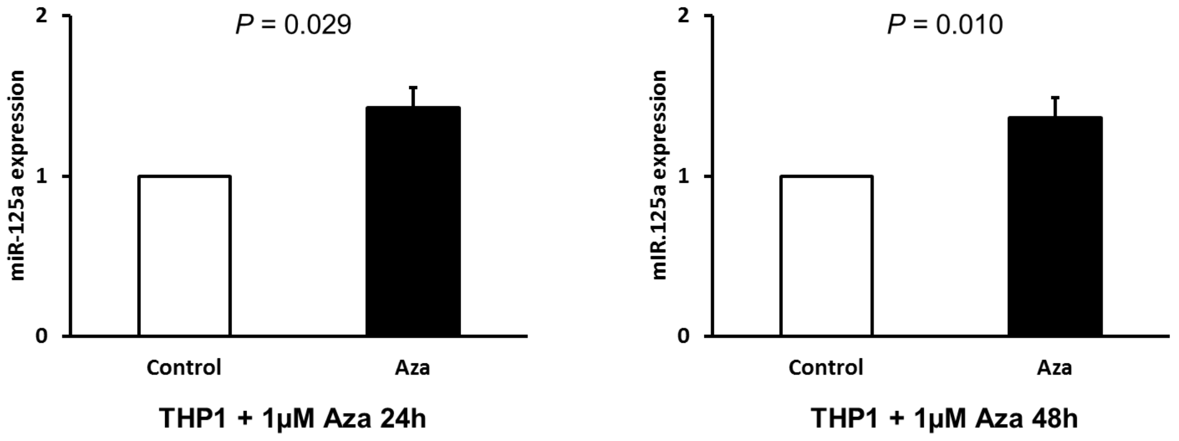

Supplementary Fig S4. miR-125a expression is induced by lower concentrations of azacitidine as well. THP1 cells were incubated with 1µM azacitidine for 24h and 48h. The relative increase of miR-125a expression in the azacitidine-treated conditions compared to controls is displayed. Graphs denote the mean +/- SD of at least three independent experiments. Comparisons against the control condition were performed using a one-sample t test against a reference value of 1. Aza, azacitidine.

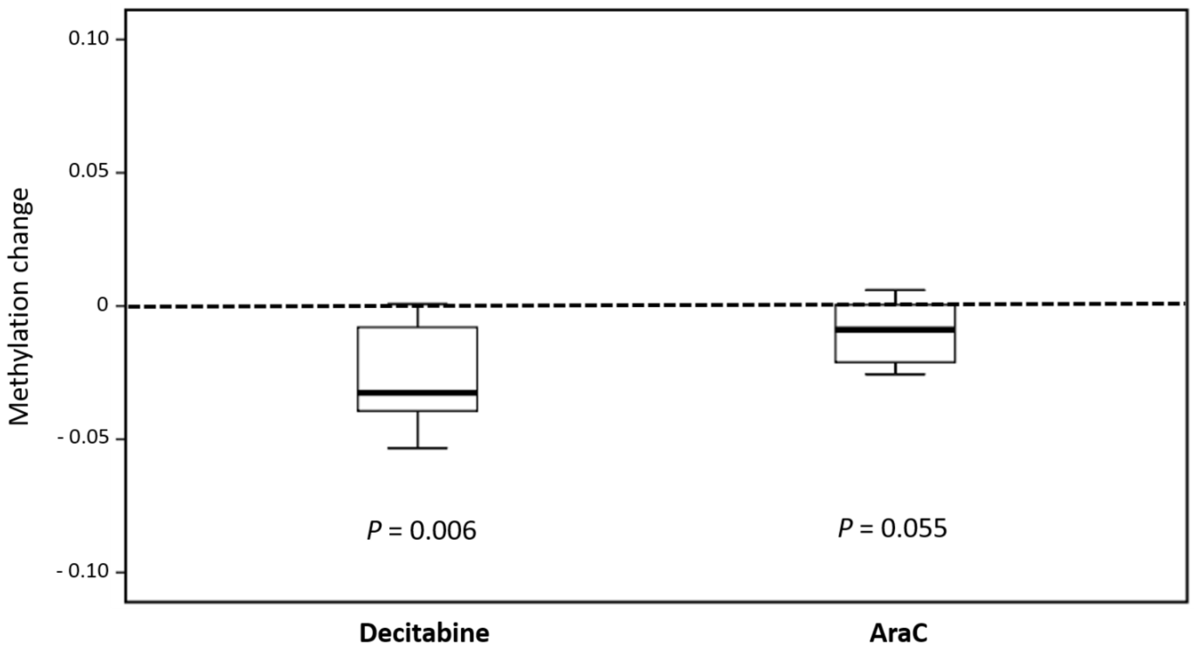

Supplementary Fig S5. The miR-125a upstream/promoter region is demethylated after HMA treatment in primary patient specimens. Database analysis of genome-wide DNA methylation profiling after treating eight primary acute myeloid leukemia (AML) patient specimens with either cytarabine (AraC) or decitabine (https://www.ncbi.nlm.nih.gov/geo/; GSE40870). In more detail, Klco et al.[2] performed genome-wide DNA methylation profiling using an Illumina HumanMethylation450 BeadChip in cytarabine/decitabine-treated specimens and compared the results to control-treated samples. Methylation of each CpG-site was displayed as methylation β-value, a value ranging from 0 to 1 (with 1 representing the maximum methylation of a CpG-site). Three CpG-sites within the upstream/promoter region of miR-125a were identified (cg25417766, cg02476580, and cg06529181); however, only one of them (cg25417766) demonstrated sufficient basal methylation for further analyses. The methylation change on the y-axis describes the change in methylation β-values in decitabine/cytarabine-treated conditions compared to the respective control-treated conditions (which are displayed by the dashed line). Comparisons against the control condition were performed using a one-sample t test against a reference value of 0. AraC, cytarabine.

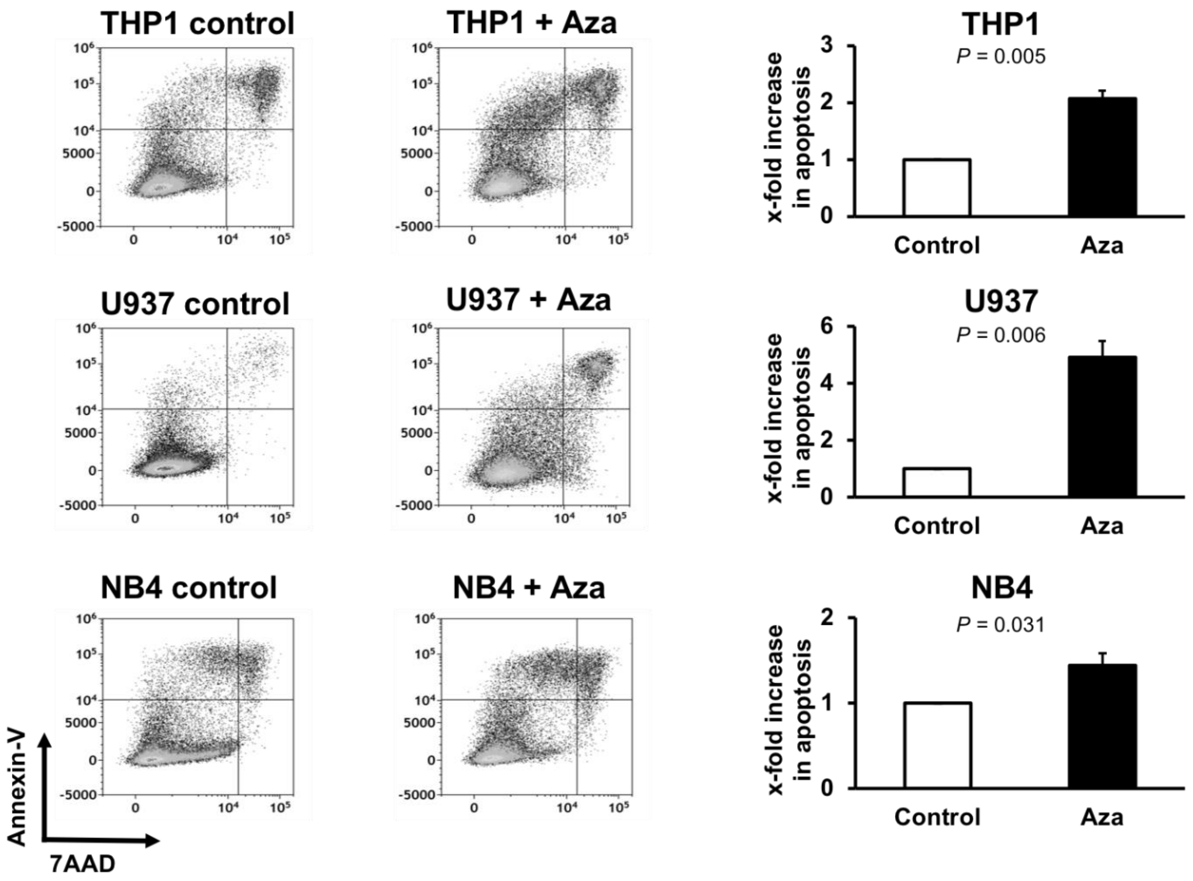

Supplementary Fig S6. Aza induces the apoptosis of myeloid cells. THP1, U937 and NB4 cells were treated with 2.5µM Aza or empty dissolvent for 24h. Subsequently, apoptosis was assessed by Annexin-V/7AAD assay. The respective control situations (treated with the empty dissolvent only) were set at a value of 1, and the relative increase of apoptosis in the Aza-treated conditions was calculated using the ratio Aza-treated to control-treated cells. Cells were considered apoptotic when they stained positive for Annexin-V or Annexin-V/7AAD. Graphs represent the mean +/- SD of three independent experiments. Comparisons against the control condition were performed using a one-sample t test against a reference value of 1. Aza, azacitidine; 7AAD, 7-Aminoactinomycin.

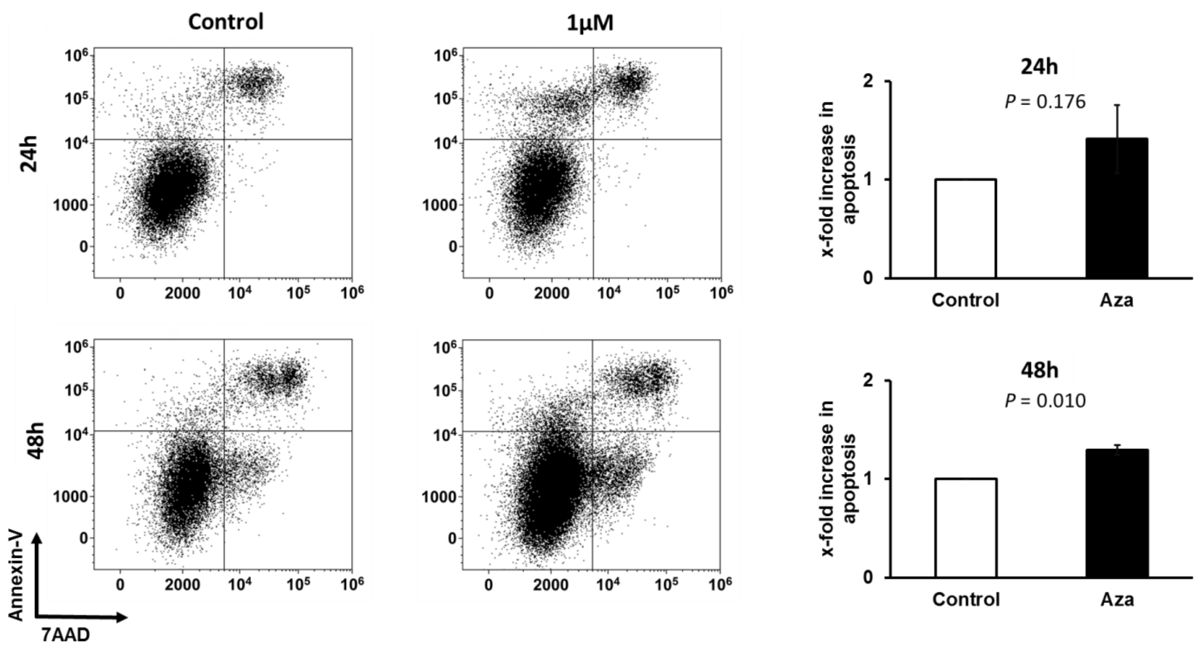

Supplementary Fig S7. Lower concentrations of Aza induce the delayed apoptosis of myeloid cells. THP1 cells were treated with 1µM Aza or empty dissolvent for 24h and 48h. Subsequently, apoptosis was assessed by Annexin-V/7AAD assay. The respective control situations (treated with the empty dissolvent only) were set at a value of 1, and the relative increase of apoptosis in the Aza-treated conditions was calculated using the ratio Aza-treated to control-treated cells. Cells were considered apoptotic when they stained positive for Annexin-V or Annexin-V/7AAD. Graphs represent the mean +/- SD of three independent experiments. Comparisons against the control condition were performed using a one-sample t test against a reference value of 1.

In combination with the data from Supplementary Fig S4, these data demonstrate that lower concentrations of Aza cause the rapid increase of miR-125a expression as well, but fail to have an immediate effect on apoptosis. Importantly, however, the effects on apoptosis are seen at a later time-point in this situation. As miR-125a overexpression causes apoptosis as well (Supplementary Figure S8), these data suggest that apoptosis in cells treated with lower concentrations of Aza might be specifically induced by the Aza-induced increase of miR-125a expression. Aza, azacitidine; 7AAD, 7-Aminoactinomycin.

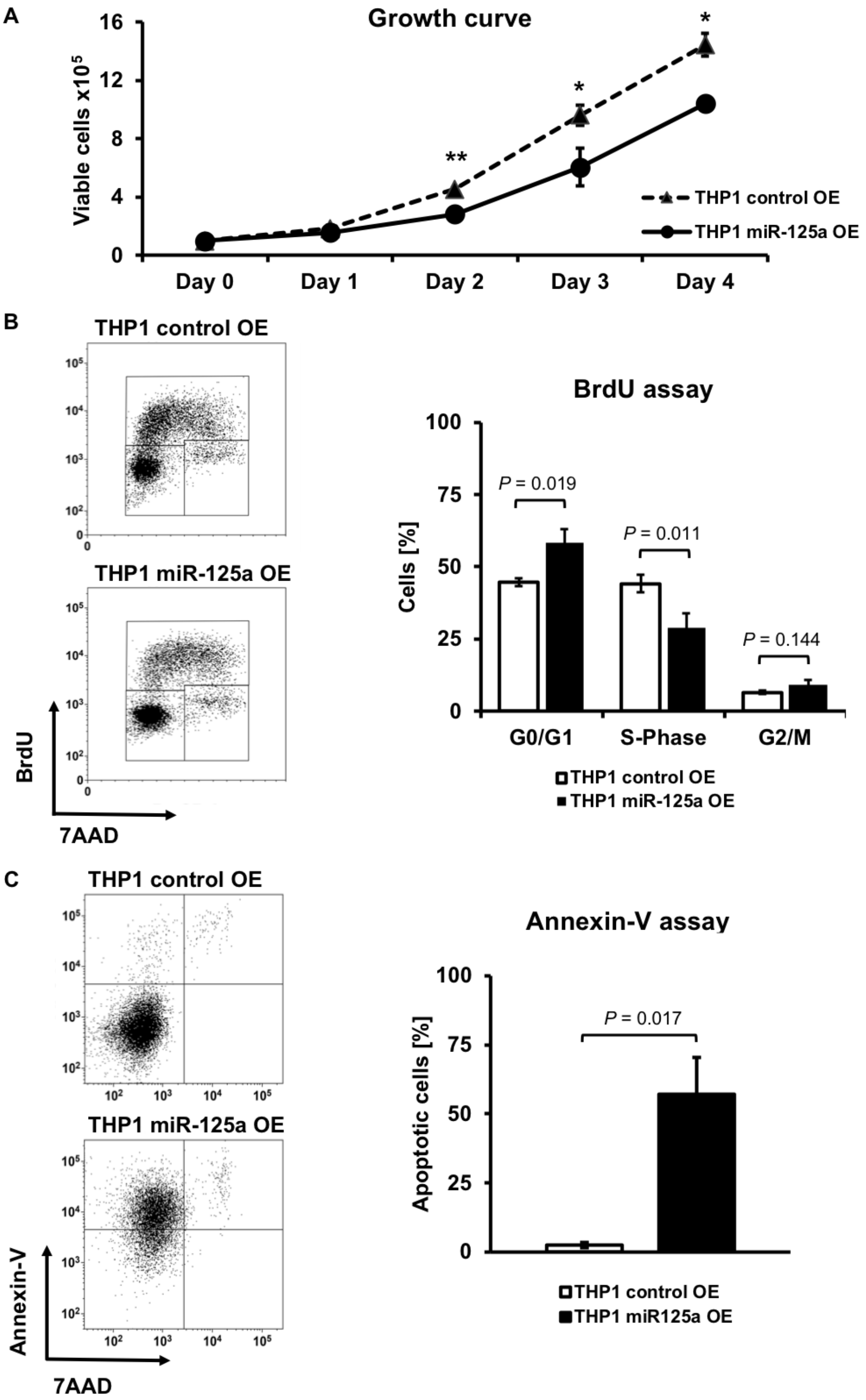

Supplementary Fig S8. In-vitro validation of the tumor-suppressive role miR-125a in monocytic leukemia cells. (A) Growth curve of THP1 miR-125a overexpressing cells (THP1 miR-125a OE) and THP1 cells transduced with the empty vector control (THP1 control OE). Cells were seeded at a density of 1x10^5/ml in serum-starved media containing 5% FBS. Subsequently, the number of viable cells was counted on four consecutive days. (B)

BrdU/7AAD cell cycle/proliferation assays were performed to assess the percentage of cells in S-phase (top gate), G0/G1-phase (left bottom gate), and G2/M-Phase (right bottom gate). (C) Annexin-V/7AAD apoptosis assays after pre-treatment with 0.5μM staurosporine for 4h. Cells were considered apoptotic when they stained positive for Annexin-V (upper left gate) or Annexin-V/7AAD (upper right gate). The graphs represent the mean +/- standard deviation (SD) of at least three independent experiments. Statistical differences were assessed by paired t test. * denotes $P < 0.050$, ** denotes $P < 0.010$. BrdU, Bromodeoxyuridine; 7AAD, 7-Aminoactinomycin.

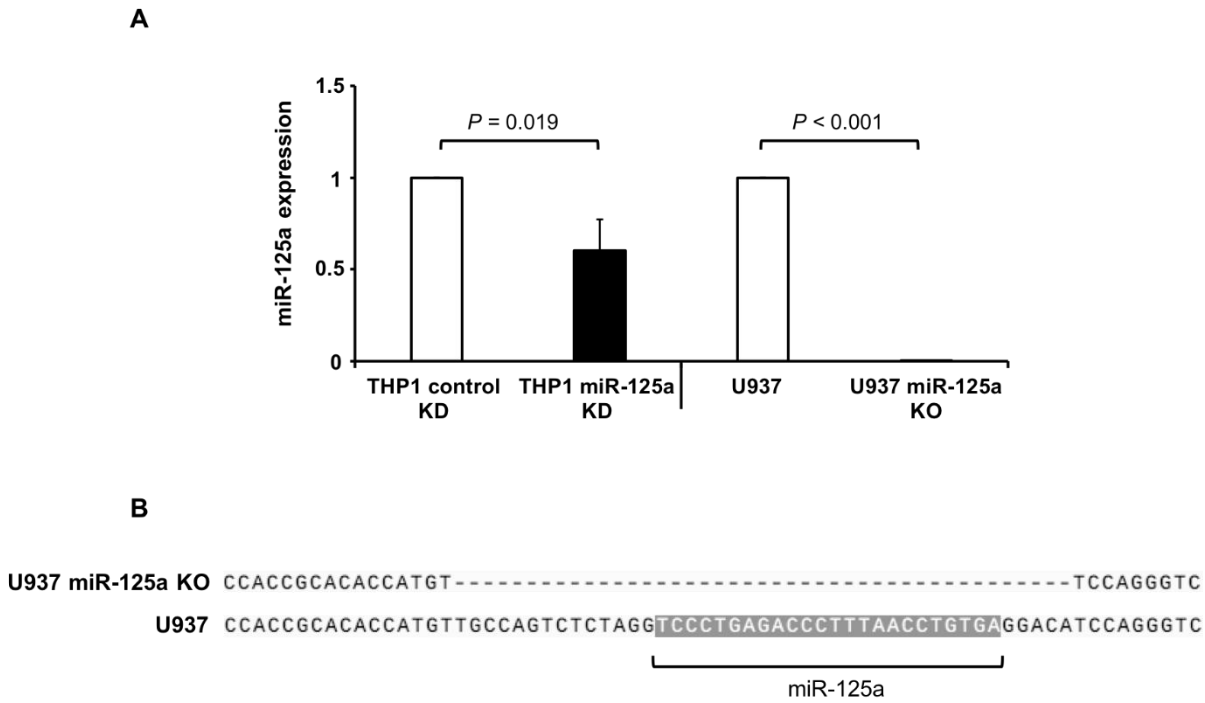

Supplementary Fig S9. THP1 knockdown of miR-125a and CRISPR/Cas9 mediated knockout of miR-125a in U937. (A) THP1 cells were transiently transfected with a miR-125a hairpin inhibitor (THP1 miR-125a KD) and miR-125a expression was assessed by qPCR three days after transfection. THP1 cells transfected with a scrambled control (THP1 control KD) were chosen as the control situation and set to a value of 1. miR-125a in U937 was deleted by the CRISPR/Cas9 methodology (U937 miR-125a KO) and miR-125a expression was controlled by qPCR. Parental U937 were chosen as the control situation and set to a value of 1. The relative expression in THP1 miR-125a KD and in U937 miR-125a KO was calculated as the ratio of the target condition to the control condition. Graphs denote the mean +/- SD of at least three independent experiments. Statistical significance was evaluated by one-sample t test against a reference value of 1. (B) Sequencing results of U937 miR-125a KO demonstrate a complete deletion of the miR-125a region on chromosome 19.

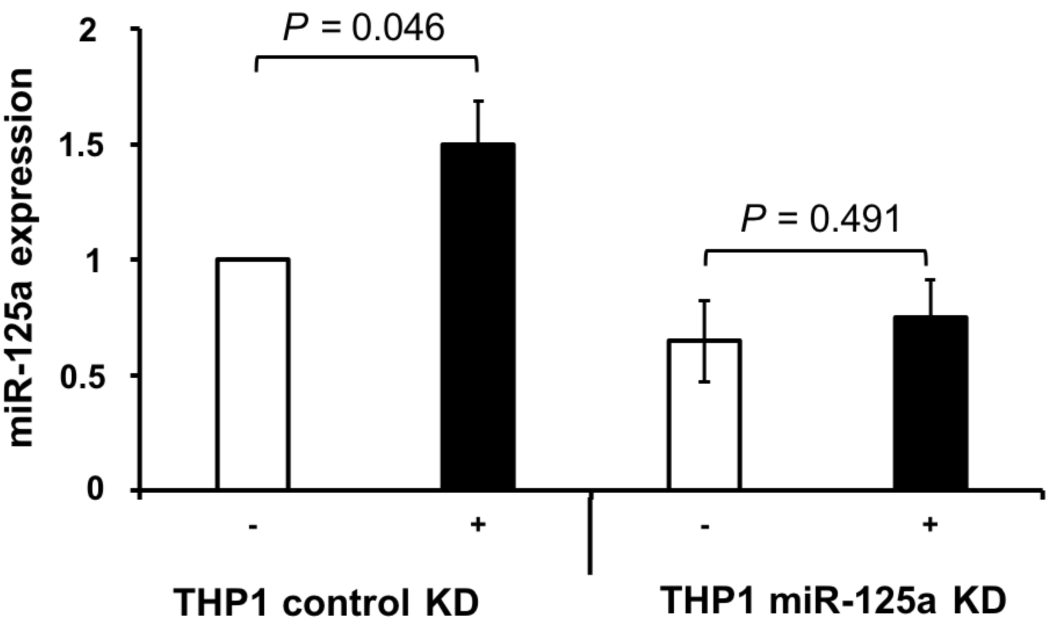

Supplementary Fig S10. The Aza-mediated increase of miR-125a expression is inhibited by miR-125a shRNA-knockdown. THP1 cells were transiently transfected with a miR-125a specific shRNA (THP1 miR-125a KD) and scrambled control shRNA (THP1 control KD), respectively. The expression of miR-125a was measured after 24h treatment with 2.5µM Aza (indicated as +) or empty dissolvent (indicated as -) by qPCR. THP1 control KD treated with empty dissolvent was chosen as the control and set to a value of 1. The relative expression in all other conditions was calculated as the ratio of the target condition to the control condition. Graphs denote the mean +/- SD of at least three independent experiments. Statistical significance was evaluated by one-sample t test against a reference value of 1 (THP1 control KD), and unpaired t test (THP1 miR-125a KD), respectively. Aza, azacitidine

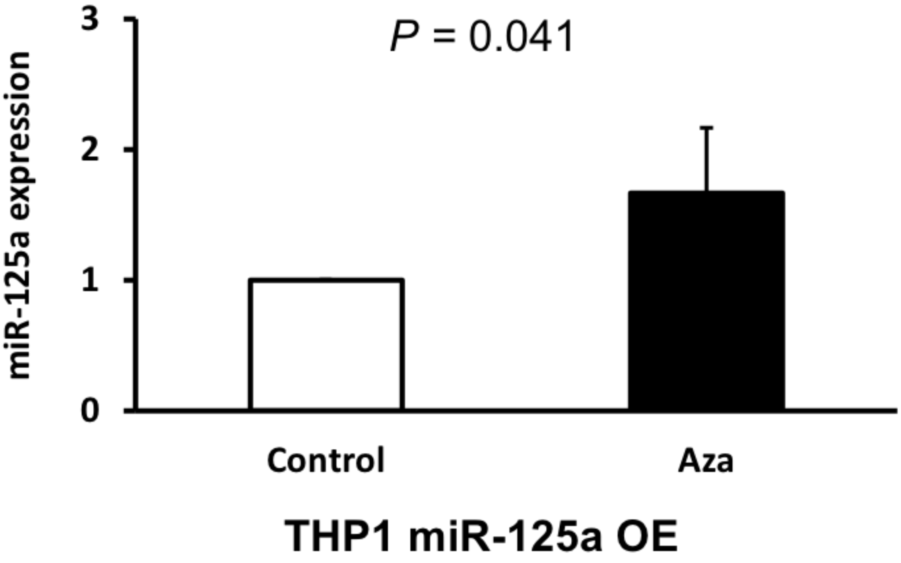

Supplementary Fig S11. Synergistic/additive effects of Aza treatment and miR-125a transduction on miR-125a expression. THP1 cells with stable overexpression of miR-125a (THP1 miR-125a OE) were treated with 2.5µM Aza. Importantly, this increased the miR-125a expression even further, which suggests a synergistic or additive effect. miR-125a expression was assessed after 24h using qPCR. THP1 miR-125a OE treated with empty dissolvent was chosen as the control and set to a value of 1. The relative expression in the other condition was calculated as the ratio of the target condition to the control condition. Graphs denote the mean +/- SD of at least three independent experiments. Comparisons against the control condition were performed using a one-sample t test against a reference value of 1. Aza, azacitidine.

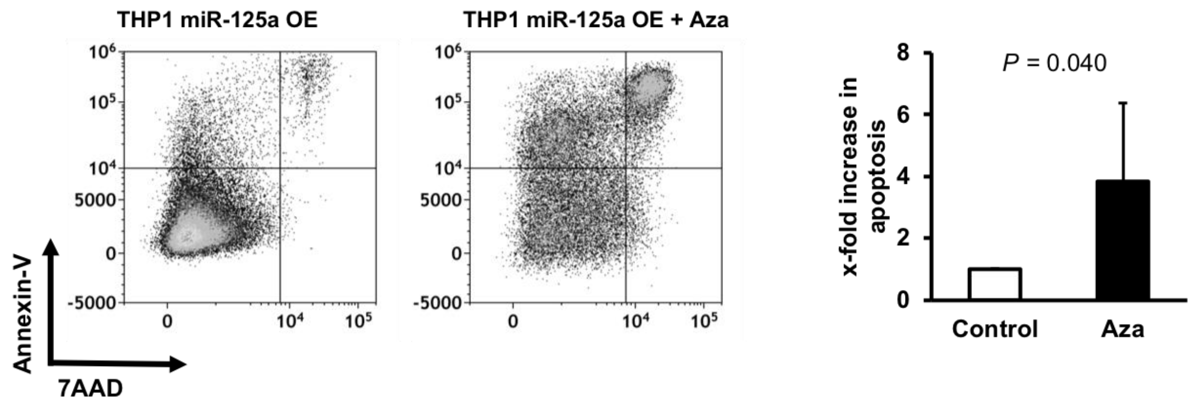

Supplementary Fig S12. Synergistic/additive effects of Aza and miR-125a overexpression on the apoptosis of myeloid cells. We demonstrated that both miR-125a overexpression and Aza-treatment induce the apoptosis of THP1 cells within this study. To reveal a potential synergism or additive effects, we treated THP1 miR-125a OE cells with 2.5µM Aza. Apoptosis was measured after 24h by Annexin-V/7AAD assay. The control condition (treated with the empty dissolvent only) was set at a value of 1, and the relative increase of apoptosis in the Aza-treated condition was calculated as the ratio of Aza-treated to control-treated cells. Cells were considered apoptotic when they stained positive for Annexin-V or Annexin-V/7AAD. Graphs represent the mean +/- SD of at least three independent experiments. Comparisons against the control condition were performed using a one-sample t test against a reference value of 1. Aza, azacitidine

A

		WBC (10^9/l)	Hb (g/dl)	Plt (10^9/l)
Wt	Mean	11.55	15.65	1216.3
	SD	2.97	0.60	696.8
Kras ^{G12D}	Mean	63.65	7.63	549.8
	SD	33.45	2.47	143.4
	P- value	**0.021**	**0.001**	**0.028**

B

C

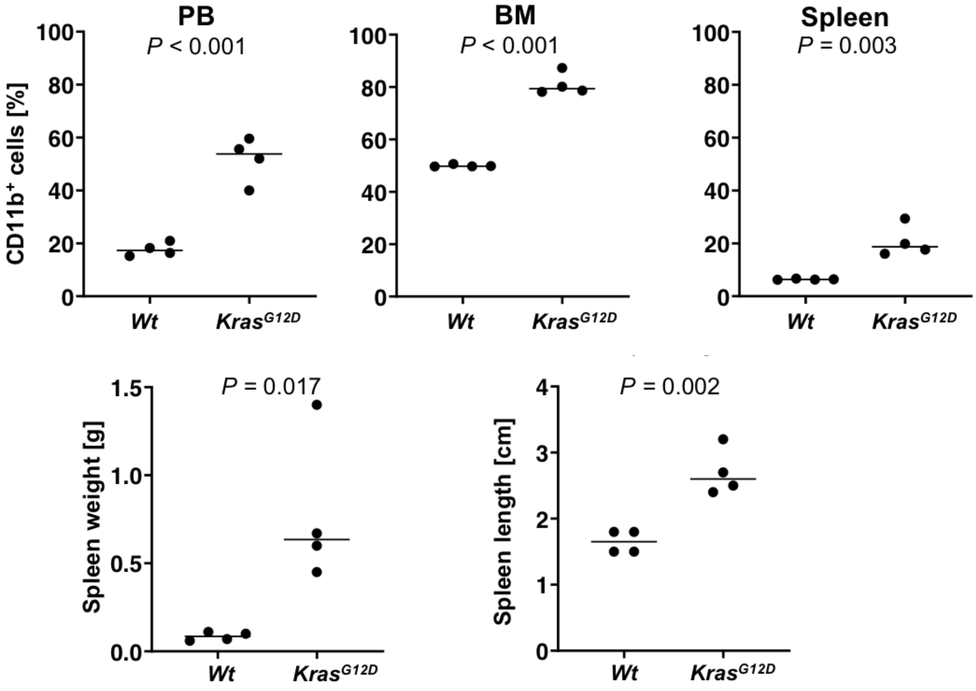

Supplementary Fig S13. CMML-like MPD is fully developed in *Mx1-Cre*$^+$/*Kras*^{G12D} mice at the time of microarray analyes. (A) Blood counts taken at the time of sacrificing for miR-expression analyses show a leucocytosis, combined with anaemia and thrombocytopenia in *Mx1-Cre*$^+$/*Kras*^{G12D} mutated mice. (B) Flow cytometry revealed that this leucocytosis was caused by an increase in myeloid cells, as evidenced by an increase of CD-11b$^+$ cells in peripheral blood, bone marrow and spleen. (C) *Mx1-Cre*$^+$/*Kras*^{G12D} mutated mice additionally presented with splenomegaly, as evidenced by an increase in both spleen weight and length. Group differences were assessed by t test. n=4 per group; the horizontal line indicates the mean. Wt, wildtype; WBC, white blood cells; Hb, haemoglobin; Plt, platelets, PB, peripheral blood; BM, bone marrow; g, grams; cm, centimetres.

Supplementary Tables

Clinical characteristics of CMML patients			Association with miR-125a expression
Gender	female	12 (33.3%)	*P* = 0.775
	male	24 (67.7%)	
Age (years)		69 (41-85)	*r = -0.24, P* = 0.156
CMML subtype	CMML-0	18 (54.5%)	*P* = 0.135
	CMML-1	9 (27.3%)	
	CMML-2	6 (18.2%)	
	NA	3	
WBC (10^9/L)		16 (2-276)	*r = 0.05, P* = 0.783
BM Blasts (%)		4 (0-19)	*r = -0.03, P* = 0.887
CMML risk score	CPSS: low	11 (36.7%)	*P* = 0.991
	CPSS: Int-1	7 (23.3%)	
	CPSS: Int-2	8 (26.7%)	
	CPSS: high	4 (13.3%)	
	NA	6	
CMML cytogenetic score	CPSS: low	22 (73.3%)	P = 0.874
	CPSS: Int	3 (10%)	
	CPSS: high	5 (16.7%)	
	NA	6	
Therapy	LDAC	1	not tested
	AZA/DEC	11	
	Ruxolitinib	2	
	7+3	4	
	alloSCT	4	
sAML transformation	no	22 (61.1%)	*P* = 0.338
	yes	14 (38.9%)	

Supplementary Table 1. Characteristics of the 36 CMML patients, and their association with miR-125a expression. Categorical parameters are presented as absolute and relative frequencies; continuous parameters are summarized using median and range (minimum – maximum). Differences in expression among patient groups formed by the categorical parameters were assessed by Mann-Whitney U or Kruskal-Wallis test. The correlation between miR-125a expression and age, WBC, and BM Blasts was analyzed with Spearman's rank correlation coefficient. NA, not available; WBC, white blood cells; BM, bone marrow; sAML, secondary acute myeloid leukemia; CMML, chronic myelomonocytic leukemia; CPSS,

CMML-specific prognostic scoring system; LDAC, low-dose cytarabine; Aza, Azacitidine; Dec, Decitabine; alloSCT, allogeneic hematopoietic stem cell transplantation.

qPCR Primer	Ordering information	
Mmu-miR-125a-5p	Qiagen, Cat# MS00001533	
Hs-miR-125a-5p	Qiagen, Cat# MS00003423	
SNORD72	Qiagen, Cat# MS00033719	
SNORD61	Qiagen, Cat# MS00033705	
RNU6b	Qiagen, Cat# MS00033740	
**Bisulfite Sequencing**	**Forward**	**Reverse**
Reaction 1	5'-AAGGGAAGAATAAATGGGAGATAT-3'	5'-ACCTAACTTCCCCCCTACCCC-3'
Reaction 2	5'-GGAGGGGAGTTAGGGAAAGT-3'	5'-ACCTAACTTCCCCCCTACCCC-3'
**sgRNA**	**Guide Sequence**	
sgRNA 1	5'-GGACCTAGAGACTGGCAACA-3'	
sgRNA 2	5'-TTAACCTGTGAGGACATCCA-3'	
**PCR Primer**	**Forward**	**Reverse**
miR-125a locus	5'-TGCTGTGTCTCTGTGGCTTC-3'	5'-GGCCAGGGGAGAAGCTAGTA-3'

Supplementary Table 2. Primer/sgRNA sequences or ordering information.

Supplementary References

1. Caraffini V, Geiger O, Rosenberger A, Hatzl S, Perfler B, Berg JL, et al. Loss of RAF kinase inhibitor protein is involved in myelomonocytic differentiation and aggravates RAS-driven myeloid leukemogenesis. Haematologica. 2020;105:375-86.
2. Klco JM, Spencer DH, Lamprecht TL, Sarkaria SM, Wylie T, Magrini V, et al. Genomic impact of transient low-dose decitabine treatment on primary AML cells. Blood. 2013;121:1633-43.
